# Multispectral Phenotyping and Genetic Analyses of Spring Appearance in Greening Plant, *Phedimus* spp.

**DOI:** 10.34133/plantphenomics.0063

**Published:** 2023-06-26

**Authors:** Taeko Koji, Hiroyoshi Iwata, Motoyuki Ishimori, Hideki Takanashi, Yuji Yamasaki, Hisashi Tsujimoto

**Affiliations:** ^1^ The United Graduate School of Agricultural Sciences, Tottori University, 4-101 Koyamacho minami, Tottori-shi, Tottori 680-8553, Japan.; ^2^ Graduate School of Agricultural and Life Sciences, The University of Tokyo, 1-1-1 Yayoi-chou, Bunkyo, Tokyo 113-8657, Japan.; ^3^ Arid Land Research Center, Tottori University, 1390 Hamasaka, Tottori-shi, Tottori 680-0001, Japan.

## Abstract

The change in appearance during the seasonal transitions in ornamental greening plants is an important characteristic. In particular, the early onset of green leaf color is a desirable trait for a cultivar. In this study, we established a method for phenotyping leaf color change by multispectral imaging and performed genetic analysis based on the phenotypes to clarify the potential of the approach in breeding greening plants. We performed multispectral phenotyping and quantitative trait locus (QTL) analysis of an F_1_ population derived from 2 parental lines of *Phedimus takesimensis*, known to be a drought and heat-tolerant rooftop plant species. The imaging was conducted in April of 2019 and 2020 when dormancy breakage occurs and growth extension begins. Principal component analysis of 9 different wavelength values showed a high contribution from the first principal component (PC1), which captured variation in the visible light range. The high interannual correlation in PC1 and in the intensity of visible light indicated that the multispectral phenotyping captured genetic variation in the color of leaves. We also performed restriction site-associated DNA sequencing and obtained the first genetic linkage map of *Phedimus* spp. QTL analysis revealed 2 QTLs related to early dormancy breakage. Based on the genotypes of the markers underlying these 2 QTLs, the F_1_ phenotypes with early (late) dormancy break, green (red or brown) leaves, and a high (low) degree of vegetative growth were classified. The results suggest the potential of multispectral phenotyping in the genetic dissection of seasonal leaf color changes in greening plants.

## Introduction

Ornamental plants have a variety of appearances based on various morphological characteristics such as color, shape, and size. These desirable morphological attributes are enhanced for exploitation depending on the purpose for which they are used. Among these, morphological characteristics such as leaf color change throughout the year due to seasonal changes of plants (phenological changes) is a highly valuable attribute especially rooftop greening. To evaluate and analyze the phenological changes in the morphological characteristics of ornamental plants, it is essential to establish a method to objectively and quantitatively evaluate the characteristics because the changes are generally continuous.

Ornamental plants are used in a variety of locations, for example, in urban greening such as rooftop greening. Greening of urban areas is considered one of the effective ways to solve the increasingly serious urban heat island phenomenon [1,2]. Rooftop greening is widely spreading especially in urban areas where the planting area is limited. In the rooftop greening, it is desirable not to use irrigation system because of its cost, water shortage in summer, and other water demand issues [3]. In addition, due to the load-bearing capacity of buildings, it is difficult to put a large amount of soil on the rooftop, and thus, it is necessary to use a thin layer of greening using a small amount of soil [4,5]. Therefore, rooftop plants must be able to tolerate environmental stresses such as drought and high temperatures and grow in a thin layer of soil without irrigation facilities [6]. Plants belonging to the family Crassuloideae, genus *Phedimus*, are perennial herbs distributed throughout the Mediterranean, Caucasus, and Asia [7]. Most of the *Phedimus* species are highly tolerant of environmental stresses such as drought and high temperature and can be grown in a thin layer of soil, making them suitable as rooftop plants. In particular, *Phedimus takesimensis* are widely used in Japan.

The genus *Phedimus* is mainly distributed in Asia and Europe, with about 20 species [8,9]. *P. takesimensis* is narrowly restricted to Ulleung Island [8]. It has been suggested that the species is monophyletic with an ancestral species of continental origin [10]. Seo et al. [10] suggest that *P. kamchaticus* and *P. aizoon* may be the ancestral species of *P. takesimensis*, and Kim et al. [8] suggest that the species *P. middendorffianus*, *P. aizoon*, *P. litoralis*, and *P. selskianus*, are likely to be the ancestral species of *P. takesimensis*. However, there are no reports on the relationship based on cross-compatibility among these species.

**Fig. 1.**
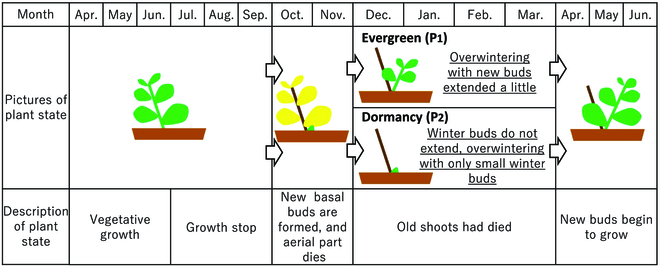
Morphological changes of plants in the genus *Phedimus* throughout the year. The upper part of the row shows the morphological changes of the evergreen species, *P. takesimensis*, while the lower part shows the morphological changes of dormant species that make up most of the wild *Phedimus* species. The parents of the F_1_ population, P_1_ and P_2_, are evergreen and dormant species, respectively. The morphological changes were observed under natural conditions in Tottori, Japan.

Since rooftop plants are also ornamentals, their appearance and color are important. Plants of this genus change their morphology with the seasons of the year (Fig. 1). In the natural environment of outdoor cultivation in Tottori, Japan, plants grow vigorously from April to June and stops growing after July. New shoots form at the base of the plants in October and November, at the same period the aging leaves wither away. Most of the wild species go dormant in winter, and the shoots overwinter in a rosette-like dormancy from December to March [7] and then break dormancy in April and start growing vigorously. Some evergreen species and cultivars overwinter with some shoots growing and leaves expanding. In this study, evergreen and dormant genotypes of *P. takesimensis* were used as parents to generate an F_1_ population.

April is the transition period from winter dormancy to spring growth. In winter-dormant wild species such as most *Phedimus* species, dormant buds, which are rosette-shaped during the winter, begin rapid internodal growth and leaf development around April. The shoots, which were red, brown, or green during winter, turn bright green after development. Since the appearance of plants is also important in rooftop greening, preferable plants have less dormancy and turn fresh green during the transition period in April. Moreover, plants with brighter green color at early growth stage are especially preferred.

Little progress has been made on genetics and breeding research of *Phedimus* plants containing *P. takesimensis*, and effective methods of morphological measurements and evaluation of genetic potential for selection are unknown. In addition, because it takes several years to evaluate the morphology of *Phedimus* plants after crossing, shortening the breeding period is also an issue. Genetic studies and studies on seasonal morphological changes such as color could contribute to the expansion of use of these plants in rooftop greening.

Plant color, an important trait for ornamental plants, is determined by the multiple wavelengths of visible light reflected from the plant body. In addition, various vegetation indices, such as normalized difference vegetation index (NDVI), are useful for evaluating plant condition, and these vegetation indices are calculated from the reflected values of visible and near-infrared (NIR) light. Multispectral image analysis can be used to evaluate these traits. The multispectral image analysis has long been used in satellite remote sensing. Multispectral satellite imagery has the advantage of covering large areas. However, its insufficient resolution makes it difficult to accurately identify plant covered areas and plant conditions [11,12]. In addition, errors in NDVI values can occur [13]. For higher-resolution measurements of plants near the ground, remote sensing has also been performed at close proximity using infrared light by a handy measuring instrument, such as Greenseeker. While this ground-based measurement method can provide high-resolution data, its drawback is that it requires a lot of human labor [14]. In recent years, measurement by unmanned aerial vehicle (UAV) equipped with multispectral cameras has been frequently used as a high-throughput plant phenotyping method to solve or at least reduce time- and labor-consuming problem [15]. Agronomic and/or physiological traits such as yield, biomass, cover ratio, and leaf area index, can be estimated nondestructively from the values of vegetation indices calculated by multispectral image analysis. This measure has been shown to be useful in estimating the extent to which plants are subjected to biotic and abiotic stresses (diseases, pests, drought, salinity, nutrient deficiencies, etc.) and in determining the health status of plants. Furthermore, it has been shown to be useful for estimating and selecting high-yielding lines and lines with high stress tolerance (disease, drought, and salinity) [16–18]. For example, studies have been reported in sorghum and wheat to predict yield by calculating plant coverage based on NDVI calculated from multispectral images [14,19]. In addition to yield and biomass estimation, some have also monitored cotton flowering in large fields by detecting floral parts from multispectral images [20].

Although multispectral cameras are considered suitable for evaluating the appearance of ornamental plants because they can measure the wavelength values of various visible light individually, few such applications have been reported so far. In this study, we investigated the usefulness of multispectral image analysis for measuring and evaluating plant body color, an important trait for ornamental plants, in order to improve breeding techniques of *Phedimus* spp. as greening plants.

In this study, we first established the measurement and evaluation methods by multispectral image analysis. Next, we clarified which of the various vegetation indices were efficient in measuring and evaluating plant color by multispectral image analysis using annual correlation. Furthermore, genetic analysis of the traits obtained by multispectral image analysis was carried out. Linkage maps and quantitative trait locus (QTL) analysis were conducted for the purpose of establishing marker-assisted breeding methods for *Phedimus* spp. in the future.

## Materials and Methods

### Generation of an F_1_ population

In this study, 2 *P. takesimensis* genotypes (Parent 1, P_1_, and Parent 2, P_2_) were used as parental lines: P_1_ is a cultivar "Tottori Fujita 1", obtained from Fujita Co., Ltd., a seed company in Japan, while P_2_ is from Gwacheon-si, Gyeonggi-do, Korea. To identify the species of P_1_ and P_2_, we sequenced internal transcribed spacers (ITSs) and compared the obtained sequences with the ITSs of the reference genome of *P. takesimensis* (National Center for Biotechnology Information database, Acc. No. MN908990). For ITS1 (295 base length), both P_1_ and P_2_ matched to *P. takesimensis*. For ITS2 (240 base length), P_1_ had one mismatch with *P. takesimensis*, while P_2_ had another mismatch with *P. takesimensis*, and thus, P_1_ and P_2_ had 2 mismatches between them. These results suggest P_1_ and P_2_ belongs to *P*. *takesimensis* or a species very closely related to it.

The 2 genotypes were used because of the contrasting features observed between them: P_1_ was less winter dormant (evergreen) and, in contrast, P_2_ was more winter dormant (dormant). Furthermore, P_1_ and P_2_ also have phenotypic differences in other traits such as leaf color, leaf size, leaf shape, and plant height. P_1_ and P_2_ were crossed in spring 2016 to generate an F_1_ segregation population. One hundred and ninety-seven seeds obtained from crosses with P_1_ as the female parent were sown to obtain 93 F_1_s. One F_1_ was obtained from a cross with P_2_ as the female parent. Many of the seeds had abnormal morphology. Seeds of the F_1_ population obtained from crosses were sown on filter paper or medium, germinated, and transferred to vermiculite. For 2 to 3 months after sowing, the plants were grown in a plant growth chamber at about 20 °C, 16-h daylength, and 3,000 to 7,000 lux. Then, the F_1_ population was fully grown. A total of 94 F_1_ plants were grown and used in subsequent studies.

Four clones (plants) of P_1_, 1 clone of P_2_, and 1 clone of each of the 94 F_1_ genotypes were planted in pots (18-cm diameter, 14-cm height) around May 2017. Horticultural soil (Cain's Corporation, Saitama, Japan) was used for planting. After planting, the plants were placed in experimental field at the Arid Land Research Center of Tottori University in Tottori, Japan (north latitude: 35.535, east longitude: 134.212), and were grown under natural conditions outdoors throughout the year. Irrigation was only applied immediately after planting. During the period of the measurement with multispectral imaging (after March 2019), no artificial irrigation was applied, i.e., irrigation was left to natural rainfall only. Changes in temperature and precipitation during the measurement period are shown in Fig. S1. The pots were spaced approximately 5 cm apart from each other and arranged in 10 vertical pots × 10 horizontal rows, with 4 individuals in the last row. All plants were grown in a 3 × 2.3 m space.

### Multispectral imaging

A multispectral camera (SIR-X2, Eva Japan Inc.) with the software for photography (Multiband Cam Client, versions 1.0.0.0 and 1.0.0.1, Eva Japan Inc.) was used for multispectral imaging. The spectrum of light reflected from a plant was captured by the multispectral camera. Nine different wavelengths (445, 500, 532, 550, 568, 676, 680, 700, and 800 nm) in the visible and NIR light regions were measured by camera imaging.

In this study, we used the multispectral camera images taken on 2019 April 16, and 2020 April 9, in Arid Land Research Center, Tottori, Japan. Photographs were taken around noon, when the sun was almost directly overhead, and the influence of sunlight was minimal. We also used cloth sunscreen (a tent ceiling) to avoid direct sunlight on the plants. The multispectral camera was set up approximately 140 cm above each plant body and imaged the plant from right above of it, and plants are taken against a white background.

### Data analysis

The imaging data was obtained as gray-scale full high-definition format video data, and the intensity of each wavelength was indicated by the gray color depth of each pixel. First of all, JPEG still images at each of the 9 wavelengths were output consecutively from moving images in the video data. From the resulting JPEG images, we manually selected 1 image for each of the plants (4 plants of P_1_, 1 plant of P_2_, and 1 plant for each of the 94 F_1_ genotypes) at each of the 9 wavelengths. The wavelength value at each pixel was calculated based on the pixel value in the selected image. Pixels with a reflection value at 800 nm (NIR light) greater than that at 680 nm (red light) were treated as plant parts, and nonplant parts such as pots and the ground were masked. The median of wavelength values of a segmented plant part was obtained and used in the succeeding analysis. MATLAB (R2019b, MathWorks) was used for data analysis. Based on the segmented plant parts, we also calculated the vegetation area of a plant. The median wavelength value was then converted to a ratio so that the sum of the 9 wavelength values equals 1. The standardized wavelength values were used for the subsequent analysis. Wavelength values were standardized to compensate for differences in light conditions due to differences in weather conditions on different measurement days. Principal component analysis (PCA) was performed based on the standardized wavelength values of all plants. Fifteen vegetation indices were calculated based on the standardized wavelength values. The 15 indices are as follows: 5 vegetation indices related to biomass, i.e., NDVI, simple ratio (SR), enhanced vegetation index (EVI), visible atmospherically resistant index (VARI), visible atmospherically resistant indices green (ViGreen); 2 vegetation indices related to plant pigments, i.e. structure insensitive pigment index (SIPI), pigment specific normalized difference (PSND); 2 vegetation indices related to chlorophyll content, i.e., chlorophyll absorption ratio index (CARI), modified chlorophyll absorption ratio index (MCARI); 3 vegetation indices related to anthocyanin, namely anthocyanin reflectance index (ARI), red green ratio index (RGRI), anthocyanin content index (ACI); 2 vegetation indices related to carotenoids, i.e. carotenoid reflectance index 1 (CRI1) and carotenoid reflectance index 2 (CRI2); and 1 vegetation index related to light use efficiency, i.e., photochemical reflectance index (PRI) (Table). Five of the 15 vegetation indices (CARI, MCARI, CRI1, CRI2, and ARI) are affected by the standardization because their formulas are not ratios. However, even in this case, the relative relationship to compare phenotypes of the 99 plants can be considered unchanged. For other vegetation indices, the values of vegetation indices are not affected by the standardization because the calculation formula is a ratio. R (version 3.6.2) was used for calculating the values of the vegetation indices [21].

**Table.** Vegetation indexes and calculation formulas analyzed in this study.

Types of vegetation indexes	Vegetation indexes	Calculation formula
Related to biomass	NDVI	(800 nm − 676 nm) / (800 nm + 676 nm)
	SR	800 nm / 676 nm
	EVI	2.5 * (800 nm − 676 nm) / (800 nm + (6 * 676 nm) − (7.5 * 500 nm) + 1)
	VARI	(550 nm − 676 nm) / (550 nm + 676 nm − 500 nm)
	ViGreen	(550 nm − 676 nm) / (550 nm + 676 nm)
Related to plant pigments	SIPI	(800 nm − 445 nm) / (800 nm − 680 nm)
	PSND	(800 nm − 445 nm) / (800 nm + 445 nm)
Related to chlorophyll	CARI	700 nm − 676 nm − 0.2 * (700 nm − 550 nm)
	MCARI	(700 nm − 676 nm − 0.2 * (700 nm − 550 nm)) * (700 nm / 676 nm)
Related to anthocyanins	ARI	(1 / 550 nm) − (1 / 700 nm)
	RGRI	676 nm / 550 nm
	ACI	550 nm / 800 nm
Related to carotenoids	CRI1	(1 / 500 nm) − (1 / 550 nm)
	CRI2	(1 / 500 nm) − (1 / 700 nm)
Related to light use efficiency	PRI	(532 nm − 568 nm) / (532 nm + 568 nm)

Intertrait annual phenotypic correlations between April 2019 and April 2020 were calculated for 9 wavelengths, principal component 1 (PC1) and 2 scores, area of plant cover, and 15 vegetation indices, and heat maps were produced. MeV software (version 4.9.0) (https://sourceforge.net/projects/mev-tm4/) was used for heat map production.

### Genotyping of genome-wide markers

Genome-wide marker data for P_1_ and P_2_ and the F_1_ population of 94 genotypes were obtained using the restriction site-associated DNA sequencing (RAD-seq) method [22]. Genomic DNA of each plant was extracted using MagExtractor -Plant Genome (TOYOBO). Detailed methods and information for library construction for the RAD-seq have been described in our previous report [23]. Genomic DNA was treated with 2 restriction enzymes, BglII and MseI. RAD-seq data (26 hundred million bp in total read average and 17 million read in average read numbers) was obtained using the Illumina HiSeq X Ten systems via paired-end sequencing (Illumina, Inc., San Diego, CA, USA). Short reads derived from RAD-Seq were analyzed using the Stacks software following the recommended setting [24].

### QTL analysis

Linkage maps were estimated using the pseudo-testcross method [25] and used in the QTL analysis. The genome-wide markers obtained with the RAD-seq were used in the linkage map. Linkage analysis was performed using the R/QTL package in R Version 3.6.2. The phenotypic values of all 27 calculated traits (area of plant cover, 9 wavelength values, PC1, 2 and 15 vegetation indices) were provided in the QTL analysis. QTL analysis was performed using the Haley–Knott regression method [26] with the composite interval mapping [27] of the R/QTL package [28]. Genotype probability calculated at every 2 cM was used in the analysis. The permutation test was performed 1,000 times. The locations of important QTLs were determined at a significance level of 5% and 10%, with the threshold of LOD value depending on the trait. Window size was done at 30.

## Results

### Generation of an F_1_ population

**Fig. 2.**
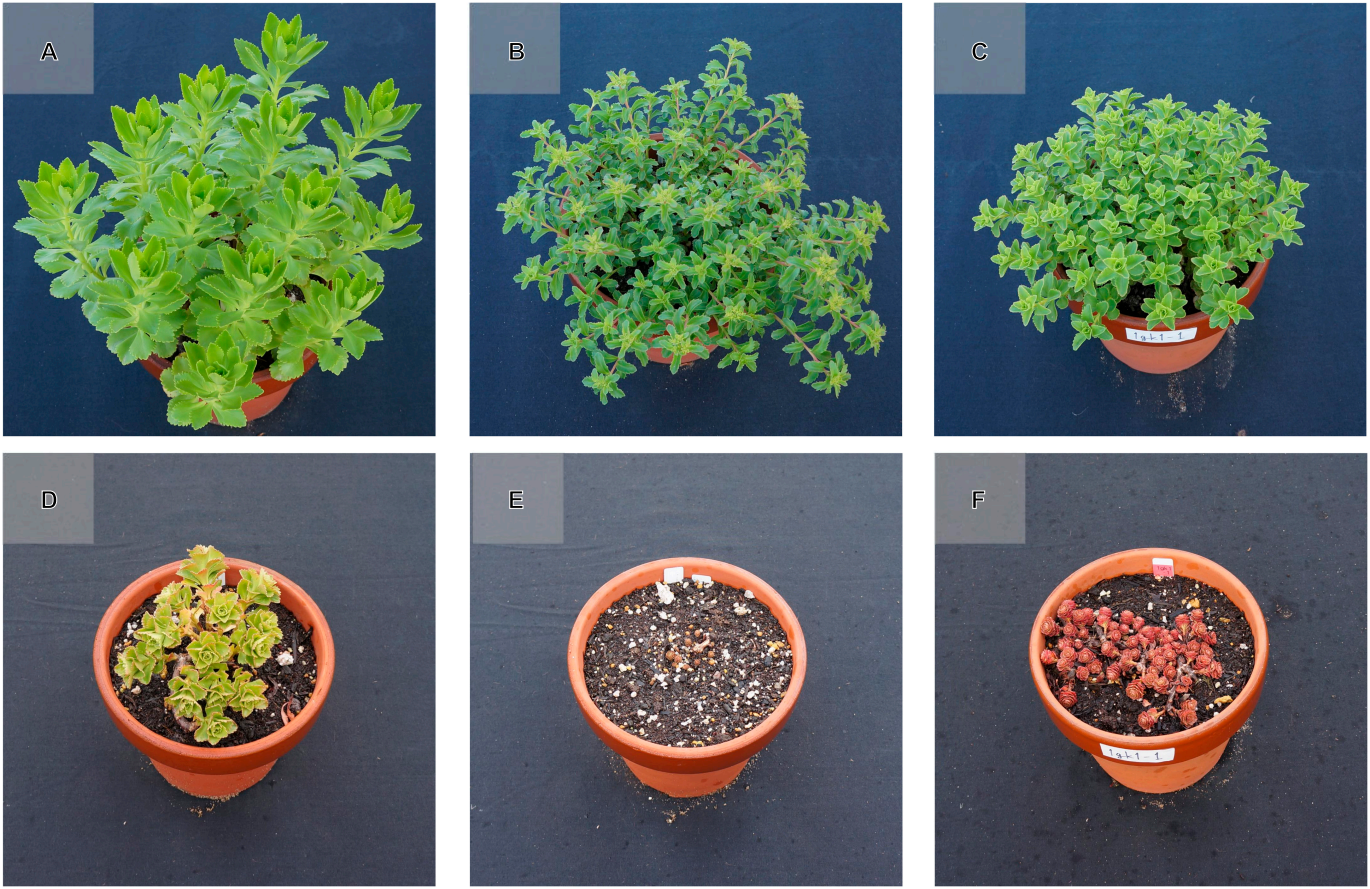
Plant appearance of P_1_, P_2_, and 1 plant of F_1_ population. The top row (A to C) was taken on 2018 May 11; the bottom row (D to F) was taken on 2018 January 9. The left vertical row (A and D) is P_1_, the middle vertical row (B and E) is P_2_, and the right vertical row (C and F) is one of the F_1_ plants. The diameter of the pot is 18 cm, and the scale of all photos is the same.

As described above, P_1_ was evergreen type, and thus, it went through the winter in the expanded leaf state with a certain degree of elongation due to the low dormancy of shoots formed at the base of the plant in autumn (Figs. 1 and 2). P_1_ had green leaves all year round. The morphological characteristics of P_1_ were large leaf size, taller plant height, thicker branches, and fewer branch number than P_2_. P_2_ was winter dormant type, and thus, new shoots that differentiate at the base of the plant in autumn were dormant and did not grow in winter. P_2_ went through the winter on rosette-shaped dormant shoots (Figs. 1 and 2). P_2_ had brown buds during the winter dormant season and had dark green leaves in spring and summer. F_1_ population has diversity in traits such as leaf color, leaf size, leaf shape, and plant height. For many phenotypes, the degree of phenotypic variability of the F_1_ population was not intermediate between P_1_ and P_2_ but rather tended toward the characteristics of P_2_. That is, for many phenotypes, such as leaf size and degree of dormancy, there appeared to be F_1_ individuals comparable to P_2_, but no F_1_ individuals comparable to P_1_. This suggest that F_1_ was segregating with heterozygous loci in P_1_ and P_2_, and many of traits in P_2_ were dominant. The F_1_ population was a segregating population, because P_1_ and P_2_ were heterozygous.

### Multispectral image capture and PCA of multispectral wavelengths

**Fig. 3.**
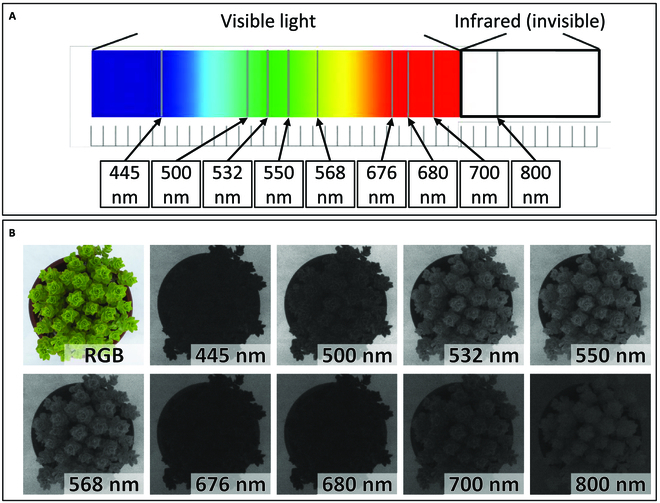
Wavelength values that can be measured with a multispectral camera, RGB images of the plant, and images of the plant at each wavelength value. The bandwidths of each wavelength are all 10 nm. Photographs are of P_1_ taken on 2019 April 9, and are all of the same individual.

**Fig. 4.**
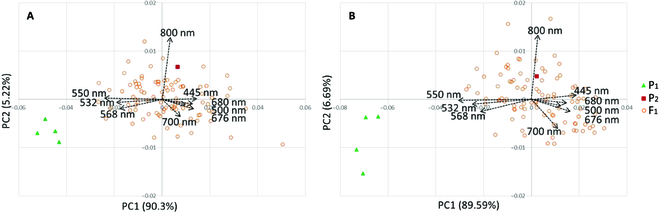
PCA of 9 different wavelength values in F_1_ segregation population in 2019 and 2020. (A and B) PCAs based on data from April 2019 and April 2020, respectively. The 4 triangles in the figure represent 4 clones of P_1_, 1 square represents 1 individual of P_2_, and 94 circles represent each of the 94 F_1_ individuals.

We obtained information of 9 different wavelength values reflected from plant bodies, based on the multispectral images (Fig. 3A and B). With PCA of the phenotypic variations in the 9 wavelengths, 2 major components that explained more than 95% of the variations were obtained. PCA in 2019 (Fig. 4A) and 2020 (Fig. 4B) showed similar trends both in the distribution of plants and in the direction of the vectors. PC1 scores of P_1_ plants were much lower than P_2_ and F_1_ plants (Fig. 4). PC1 explained the contrast between the reflection of green visible light (532, 550, and 568 nm) and the one of the other (blue and red) visible light (at 445, 500, 676, 680, and 700 nm). The value of this PC1 was high at about 90% in both years. PC2 scores of P_1_ plants were slightly lower than or same as P_2_ and F_1_ plants. PC2 explained the reflection of NIR light (invisible light) at 800 nm. The variation explained by PC2 was much lower than PC1, ranging from 5% to 7% of the total variation in both years. The F_1_ plants dispersed but were located in the same cluster as the P_2_ plant, while the cluster of P_1_ plants was separated from the cluster of P_2_ and F_1_, suggesting that P_1_ was distinguished from the F_1_ population by its high reflection of green wavelengths and high absorption of blue and red.

### Relationship between plant color and area of plant cover

**Fig. 5.**
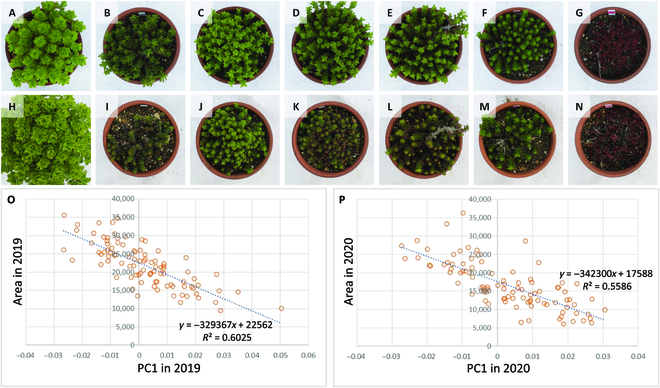
Plant appearance of P_1_, P_2_, and F_1_, and correlation between PC1 and area of plant cover. (A to G) Plant appearance photographed on 2019 April 16 and (H to N) on 2020 April 9. The 2 individuals in the upper and lower rows are identical. (A) and (H) are P_1_, (B) and (I) are P_2_, and the rest are F_1_ individuals. The F_1_ individuals in (C) to (G) have quartiles of PC1 values of 0%, 25%, 50%, 75%, and 100%, respectively. The diameter of the pot is 18 cm, and the scale of all photos is the same. (O and P) The correlation between PC1 and covered area in 2019 and 2020, respectively. The horizontal axis shows the value of PC1, and the vertical axis shows the clothing coverage. The 94 circles in the figure represent each of the 94 F_1_ individuals.

To check for morphological differences due to differences in PC1 scores, quartiles were calculated among the F_1_ population for PC1 values in 2019. The plant appearance of F_1_ individuals with quartiles of 0%, 25%, 50%, 75%, and 100% are shown in Fig. 5C to G. The plant appearance of these F_1_ individuals and the same individuals in 2020 is shown in Fig. 5J to N. The plants of P_1_ (Fig. 5A and H) and P_2_ (Fig. 5B and I) are also shown.

To check the relationship between plant color and area of plant cover, we calculated the correlation between the value of PC1 and the value of covered area in all F_1_ individuals (Fig. 5O and P). There was a negative correlation between the value of PC1 and the value of covered area in both 2019 and 2020. The correlation for 2019 is *R*^2^ = 0.60 and for 2020 is *R*^2^ = 0.56, which is quite high for both years. The lower the value of PC1, the larger the covered area, and conversely, the higher the value of PC1, the smaller the covered area. It was significant at *P* ≤ 0.01 in both 2019 and 2020.

### Annual phenotypic correlations in plant color and vegetation indices

**Fig. 6.**
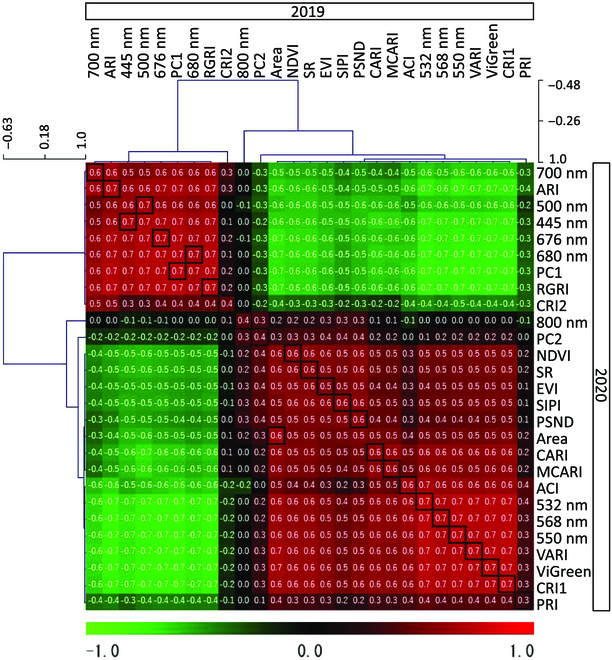
Annual correlation of all traits between April 2019 and April 2020. The horizontal axis shows all traits in 2019 and the vertical axis shows all traits in 2020. All traits are the area covered, 9 wavelength values, PC1 and 2 values, and 16 vegetation indices. The numbers in the cells indicate *R*-values.

Green wavelengths (532, 550, and 568 nm), which were negatively related to PC1 scores, and vegetation indices related to biomass, chlorophyll, and plant pigments, and area covered were positively correlated with each other (Fig. 6). Visible light other than green (445, 500, 676, 680, and 700 nm), which were positively related to PC1 scores, and vegetation indices related to anthocyanins other than ACI were positively correlated with each other. There was a negative correlation between the former set and the later set of variables.

The coefficient of the annual phenotypic correlations of the total 27 traits were larger than 0.7 in 13 traits and larger than 0.4 in 26 traits. In PC1, the annual correlation was high at 0.73, being the second highest correlation value of the 27 traits. Also, in the vegetation indices related to biomass (VARI and ViGreen) and anthocyanin (ARI and RGRI), the annual correlation was high (around 0.7). In the wavelength value of 800 nm and PC2, which explained the reflection at 800 nm, the annual correlation was not so high (around 0.4).

### RAD-seq and QTL analysis

**Fig. 7.**
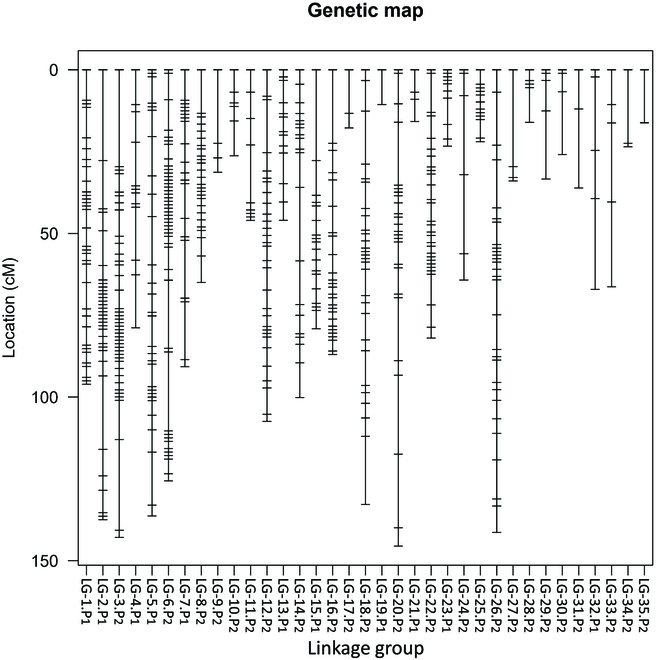
Genetic map.

The linkage map consisted of 35 linkage groups (11 linkage groups from P_1_ and 24 linkage groups from P_2_) (Fig. 7). The total chromosome length was 2,370.14 cM, and the average length of chromosomes was 67.72 cM. The total number of markers was 549, and the average distance between markers was 4.61 cM.

**Fig. 8.**
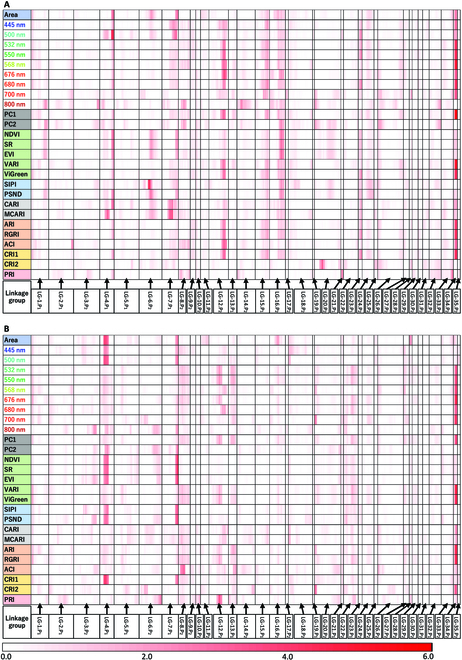
Linkage group positions showing high LOD values of QTLs for each trait. (A and B) The results of QTL analysis for all traits on 2019 April 16, and 2020 April 9, respectively. The vertical axis represents traits, and the horizontal axis represents linkage groups. Higher LOD values are shown in darker red. In 2019 (A), a QTL significant at the 5% level was detected at the same position on LG-34.P_2_ in 8 traits: 532, 550, and 568 nm and PC1, VARI, ViGreen, RGRI, and CRI1. The QTL was marginally significant (at the 10% level) in 680 nm and ARI. In 2020 (B), the QTL on LG-34.P_2_ was detected in 6 traits, 676 and 700 nm and VARI, ViGreen, ARI, and RGRI, at the 5% level. The QTL was marginal significant (at the 10% level) in 550 and 568 nm. The QTL on LG-7.P_1_ was significant at the 5% level in NDVI, SR, and EVI.

In April 2019, a significant QTL was observed on a linkage group, the P_2_-derived linkage group 34 (henceforth referred to as LG-34.P_2_) (Fig. 8A). The QTL detected on LG-34.P_2_ was significantly associated with 8 traits : 532, 550, and 568 nm and PC1, VARI, ViGreen, RGRI, and CRI1, at 5% level of probability. In addition to the above 8 traits, this QTL was marginally significantly associated at the 10% level with 2 more traits: 680 nm and ARI. The vegetation indices in which the QTL was detected significantly were related to biomass, anthocyanins, and carotenoids. Among all the traits, PC1 had the highest LOD value (LOD = 5.7) (Fig. S2A and Table S1).

In April 2020, a QTL was detected at the same position of LG-34.P_2_ as in April 2019 (Fig. 8B). In 2020, the QTL was significantly associated with 6 traits: 676, 700 nm and VARI, ViGreen, ARI, and RGRI, at the 5% significance level. In addition to the above 6 traits, this QTL was marginally significantly associated (at the 10% level) with 2 more traits: 680 nm and ARI. The vegetation indices in which the QTL was detected significantly were related to biomass and anthocyanin. Among all the traits, the LOD value of VARI, one of vegetation indices related in biomass, was the highest (LOD = 4.7) (Fig. S2B and Table S1). The QTL on LG-34.P_2_ was detected both in April 2019 and April 2020 at the 5% level for VARI, ViGreen, and RGRI.

In April 2020, a QTL was detected on P_1_-derived linkage group 7 (henceforth referred to as LG-7.P_1_) (Fig. 8B). For QTL in LG-7.P_1_, LOD values were at 5% level for 3 traits: NDVI, SR, and EVI. QTL were found in vegetation indices related to biomass. Among all the traits, the LOD value was highest in SR, which is a vegetation index related to biomass, with a LOD value of 4.5 (Fig. S2C and Table S1). The QTL on LG-7.P_1_ was detected in April 2020 but not in April 2019.

**Fig. 9.**
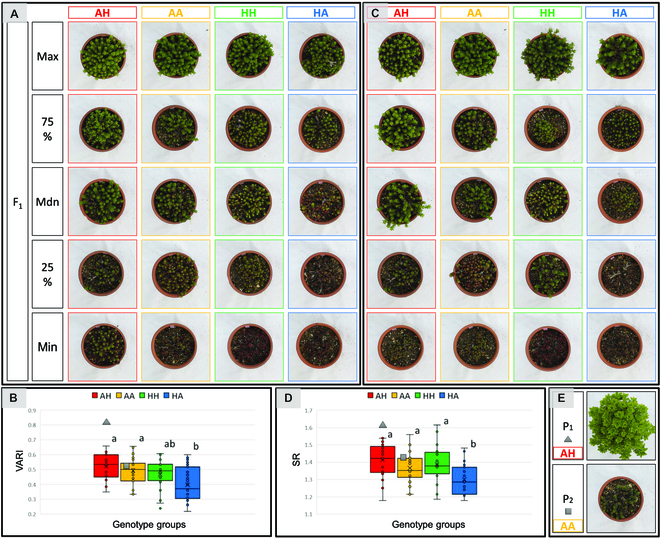
Plant appearance of representative F_1_ within each genotype group on 2020 April 9. Representative F_1_ individuals with quartiles of 100%, 75%, 50%, 25%, and 0%, respectively, within each genotype group of F_1_ for VARI values for which QTL were found in LG-34.P_2_ (A). The values of VARI among each group were also compared (B). Similarly, representative F_1_ individuals were also shown for SR values where QTL was observed in LG-7.P_1_ (C), and SR values among each group were compared (D). Letters in (B) and (D) indicate *P* ≤ 0.01 by ANOVA. Plant appearance of P_1_ and P_2_ on the same measurement date are also shown (E).

Two QTLs on LG-34.P_2_ and LG-7.P_1_ were detected simultaneously in April 2020 associated with multiple traits. We grouped F_1_ individuals into 4 classes of genotypes with markers close to the QTLs (Fig. 9). Based on the genotypes of the marker closest to the QTL in LG-34.P_2_, the F_1_ individuals were grouped into A (homozygous) or H (heterozygous). Similarly, the F_1_ individuals were grouped into A or H based on the genotype of the marker closest to the QTL of LG-7.P_1_. Based on the genotypes of the markers, the F_1_ individuals were grouped into 4 classes: AH, AA, HH, and HA, where the first capital letter represents the genotype of the QTL of LG-34.P_2_.

In April 2020, we compared the values of VARI, which had the highest LOD value in the QTL of LG-34.P_2_ (Fig. 9A and B), and SR, which had the highest LOD value in the QTL of LG-7.P_1_ (Fig. 9C and D), because there was no trait in which these QTLs were detected both at LG-34.P_2_ and LG-7.P_1_. For each trait value, the quartiles within each of the 4 genotype groups were calculated, and representative F_1_ individuals were selected from each genotype group (Fig. 9A and C).

One-way analysis of variance (ANOVA) showed that there was a significant difference in the value of VARI for the QTL at LG-34.P_2_ between the HA group and the 2 groups (AH and AA) at *P* ≤ 0.01 (Fig. 9B). There was a significant difference in SR of the QTL identified in LG-7.P_1_ between HA and the other 3 groups (AH, AA, and HH) at *P* ≤ 0.01 (Fig. 9D). There was a difference in morphological plant status among the groups where statistically significant differences were observed. For the QTL identified in LG-34.P_2_, the AH and AA groups tended to have higher values of VARI, earlier dormancy breaking of shoots, advanced internodal growth and leaf development, and brighter green leaves. In contrast, the HA group tended to have lower VARI values, less advanced dormancy breaking, and red or brown leaves similar to those in the dormant phase (Fig. 9A). The same trend was observed for the SR values of the QTL in LG-7.P_1_ (Fig. 9C).

The genotype of P_1_ belonged to the AH group. The values of VARI and SR of P_1_ were much higher than those of the F_1_ population. Visually, P_1_ had the most flourishing branches and leaves (Fig. 9B, D, and E).

## Discussion

We crossed P_1_ and P_2_ to obtain an F_1_ hybrid population. The morphological characteristics (degree of dormancy, leaf size and shape, plant height, number of branches, etc.) of F_1_ individuals were closer to P_2_ in most cases. The low level of dormancy of dormant buds in winter, i.e., evergreenness in winter, is a trait of importance in *Phedimus* species including *P. takesimensis*. The F_1_ population had characteristics similar to P_2_ that were highly dormant. Although the genetic background of P_1_ and P_2_ is not clear, the results suggest that the alleles harbored by P_2_ of genes related to dormancy are dominant. In the future, backcrossing and sibling crosses using F_1_ may allow the dissection of useful characteristics of P_1_ that did not directly appear in the F_1_ population analyzed in this study.

In the PCA (Fig. 4), PC1 reflects mainly the variations in visible light, which means that it represents the visible leaf color of the plant. When we compared the photos of representative F_1_ plants in Fig. 5C to G and J to N, the leaves of plants with higher PC1 values were redder and those with lower PC1 values were greener. The fact that the contribution of PC1 in PCA was quite high, about 90%, indicates that the variations observed in F_1_ can be mostly explained by the visible leaf color, suggesting the multispectral image analysis can capture the variations and is suitable for the evaluation of the variations. Most of the plant measurement studies using multispectral imagery reported so far have measured large plots with a multispectral camera mounted on a UAV. There have been reports of estimating the state of plants by vegetation indices calculated from wavelength values and estimating the extent of disease damage suffered by crops and the effects of fertilizer application [29,30]. In this study, measurements were made by proximity, and in addition to evaluating vegetation indices, analysis using visible light values was attempted, and it was found that the visible colors of plants could be successfully evaluated. We were able to show that multispectral imaging is also effective in evaluating visible color by this method.

One of the scientific importance of this study is that by using multispectral analysis of leaf color, we were able to capture not only variation in leaf color but also dormant genetic variation manifested in leaf color variation. When studying phenotypic variation in organisms, it is important not only to measure the phenotypic variation but also to clarify the genetic factors controlling the variation and the environmental variation that cannot be explained by genetic factors. In the case of plant dormancy, which was the subject of this study, it is important not only that a method for measuring leaf color variation has been developed but also that the variation measured by this method has been shown to be explained by genetic factors such as QTL. Through this study, we were able to propose a new measurement method for genetic analysis of plant dormancy.

For seed dormancy, there is 1 study using multispectral analysis [31]. In most studies, plant dormancy was evaluated visually only, as in QTL analysis for budding time in apple and peach and QTL analysis for fall dormancy in alfalfa [32–34]. Exceptions include studies using NDVI values obtained with the GreenSeeker instrument in switchgrass and wheat [35,36]. Multispectral remote sensing assessments of seasonal dormancy have been reported for forest canopy and tallgrass prairie [37,38]. It has been reported that changes in plant growth activity due to water stress and aging were captured by vegetation indices calculated from multispectral camera measurements and contributed to QTL discovery [39–41]. Similarly, in this paper, we were able to capture plant growth activity during dormancy breakthrough and find QTL related to it. However, to date, no QTL analysis of plant dormancy has been performed using individual-based measurements and multispectral analysis.

The quantitative evaluation of leaf color using multispectral imaging has improved the objectivity of the data and the information content of the data obtained, providing multidimensional quantitative measurements. Multispectral analysis allows evaluation of the plant body not only at a limited number of wavelengths and indices, such as RGB or NDVI, but also multivariate using PCA or based on different vegetation indices, such as in this study. This allowed us not only to detect QTLs but also to identify differences in the characteristics of each QTL, such as differences in the associated wavelengths and vegetation indices. We were also able to identify differences in traits, for example, a important QTL for leaf color variation is related to anthocyanins, which are strongly associated with dormancy.

Color chart evaluation and color reader can be used as leaf color evaluation methods. In the former method, even if a color chart showing continuous color change is used, it is a 1-dimensional class value, and the amount of information is much lower than the multidimensional quantitative measurements obtained by multispectral analysis. In addition, there is a problem with objectivity (i.e., nonsubjectivity), because color chart evalutaion depends on the subjectivity of the evaluator. Although a color reader can be used to obtain highly objective quantitative measurements, it is difficult to determine representative measurement points when there is color variation throughout the plant body.

In April, when the measurements were taken, dormancy breaking occurs. Most wild species of *Phedimus* plants, including *P. takesimensis*, P_2_ and F_1_, have red, brown, or dark green rosette-shaped dormant buds in winter, but when dormancy is broken and vigorous elongation begins, the leaf color changes to bright green and the area covered increases. There was a negative correlation between the PC1 value and the covered area (Fig. 5O and P), suggesting that PC1 indicates not only the visible leaf color but also the covered area and acceleration of dormancy breakage.

To the best of our knowledge, this study is the first report of linkage mapping and QTL analysis in *P. takesimensis* species. Recent studies have provided insights into the phylogenetic relationships in *P. takesimenseis* [10,42]. Few genetic studies have been done on *Phedimus* plants including *P. takesimensis*. However, an important trait for horticultural plants, leaf color, has not been analyzed yet. The present study provides insight into the genetic analysis of leaf color and dormancy breaking in *P. takesimensis*. The results obtained in this study will be useful for further genetic dissection of the leaf color and dormancy breaking characteristics of this species.

Two QTLs, in LG-34.P_2_ and LG-7.P_1_, were found simultaneously in April 2020, and QTLs were found for many of the vegetation indices related to biomass and anthocyanin among the traits. The comparison of these 2 QTLs among the genotype groups of the F_1_ population showed statistical differences. In addition, there were visual differences in the degree of vegetative growth and leaf color among the genotype groups. One genotype group tended to have red leaves, a low degree of flourishing, and a slow dormancy breaking, while the other group tended to have green leaves, a high degree of flourishing, and a fast dormancy breaking. Therefore, we speculated that these 2 QTLs are responsible for the early or late dormancy breaking. By using these 2 QTLs, we may be able to select plants that break dormancy early and have well-developed and bright green leaves during the transition period in spring.

Of the 2 QTLs, the LG-34.P_2_ QTL was common between the 2 measurement dates across both years. Furthermore, the QTL was found to have pleiotropic effect on multiple traits, and its significance probability level lower than 5% level for most of the traits. This suggests that the QTL of LG-34.P_2_ had a relatively large effect in leaf color and was important for the genetic improvement of the leaf color.

Since the *R*-values of the annual correlations (Fig. 6) were high for many traits, it can be said that there is reproducibility between the results of 2019 and 2020. In particular, the *R*-values of the 8 visible light wavelength values except 800 nm and the *R*-value of PC1 were both high (0.6 or more), suggesting that the visible characteristics of leaf color can be evaluated reproducibly by the multispectral camera.

The wavelength value of 800 nm had a relatively low *R*-value of 0.4 for the annual correlation (Fig. 6). The *R*-values of VARI and ViGreen among the vegetation indices related to biomass and ARI and RGRI among the vegetation indices related to anthocyanin were relatively high because 800 nm was not used in their calculations. To evaluate the leaf color variations in this species, it may be better to use these vegetation indices that do not use 800 nm in the calculation for better reproducibility. Anthocyanins represented by ARI and RGRI are known to have absorption maxima in the wavelength region of 510 to 550 nm [43] while chlorophyll a by VARI and ViGreen absorbed at 676 nm [44]. Since these anthocyanin and chlorophyll indices were negative correlations in F_1_ populations (Fig. 6), amounts of these pigments are negatively associated. As similar, these relationship were observed in angiosperm evergreen species of acyanic (winter-green) and anthocyanic (winter-red) [45].

The QTL identified in LG-7.P_1_ was only detected in April 2020 and not in April 2019. In April 2020, 3 traits, NDVI, SR, and EVI, had QTL in LG-7.P_1_, but the wavelength value of 800 nm was used to calculate these 3 vegetation indices (Table). The use of the 800-nm wavelength value, which has a low annual correlation, in the calculation of the vegetation indices may be one of the reasons why the QTL in LG-7.P_1_ was only obtained on one measurement date. In addition, April is the time of year when *Phedimus* plants including *P. takesimensis* undergo rapid internodal growth of dormant buds to break dormancy, and morphological changes are particularly large. Therefore, the QTL in LG-7.P_1_ may have been obtained only on one of the measurement dates due to a small time series variation in growth environment such as temperature. The QTL for LG-34.P_2_ was common on the 2 measurement dates in 2019 and 2020, suggesting that it is highly reproducible.

It is known that NIR reflectance values are correlated with leaf structure, such as cuticle layer thickness [46]. The time of year when the measurements were taken was a time of rapid internode and leaf growth, and the leaf structure was changing dramatically, which may also have facilitated the annual differences in the 800-nm reflectance values.

The traits whose QTLs were represented in LG-34.P2 were vegetation indices related to biomass (VARI and ViGreen) and anthocyanins (ARI and RGRI). These used 550 nm in their calculations; 550 nm is the absorbance of anthocyanins [43]. Anthocyanins are known to accumulate in *Galax urceolata* and other species in winter to protect leaves from light damage caused by low temperatures, and to disappear in spring when temperatures rise [47]. The LG-34.P2 QTL may be involved in this anthocyanin mechanism. The traits whose QTLs were represented in LG-7.P1 were vegetation indices related to biomass (NDVI, SR, and EVI). These were calculated at 800 nm. NIR reflectance values are known to correlate with leaf structure, such as the thickness of the cuticle layer [46], and the QTL in LG-7.P1 may be related to these structural changes in the cuticle layer for the growing season.

The multispectral image analysis used in this study revealed that the quantitative evaluation and genetic analysis of phenotypes such as plant color at the time of dormancy breakage are possible. The high contribution of PC1, which represents visible light in Fig. 4A and B, indicates that this method is particularly suitable for quantitative evaluation of visible color in ornamental plants. In addition, since there was a relationship between plant color and dormancy (Fig. 5), this method can appropriately capture the state of plants during the period of breaking dormancy, which is a time when the color and size of plants gradually change. Since there are few examples of multispectral image analysis of ornamental plants, the new findings of this study are very important. The method may be applicable to other perennial ornamental plants as well.

## Data Availability

The data could be given upon reasonable request from the corresponding author.

## Supplementary Materials

Supplementary 1 Table S1. Summary of the QTLs detected in this study. Fig. S1. Weather information during the measurement period. Fig. S2. The LOD value of the trait with the highest LOD value on each measurement day and at each QTL.
