# Supplementary material for: Multispectral Phenotyping and Genetic Analyses of Spring Appearance in Greening Plant, Phedimus spp
Source: Plant Phenomics. 2023 Jun 26;5:0063. doi: 10.34133/plantphenomics.0063 (PMC10292581; doi:10.34133/plantphenomics.0063)
Supplement: Supplementary 1 — Table S1. Summary of the QTLs detected in this study. Fig. S1. Weather information during the measurement period. Fig. S2. The LOD value of the trait with the highest LOD value on each measurement day and at each QTL. [file plantphenomics.0063.f1.docx]

Supplementary Materials

**Supplementary Table**

**Supplementary Table 1. Summary of the QTLs detected in this study.**

PEV phenotypic variance explained.

**Supplementary Figures**
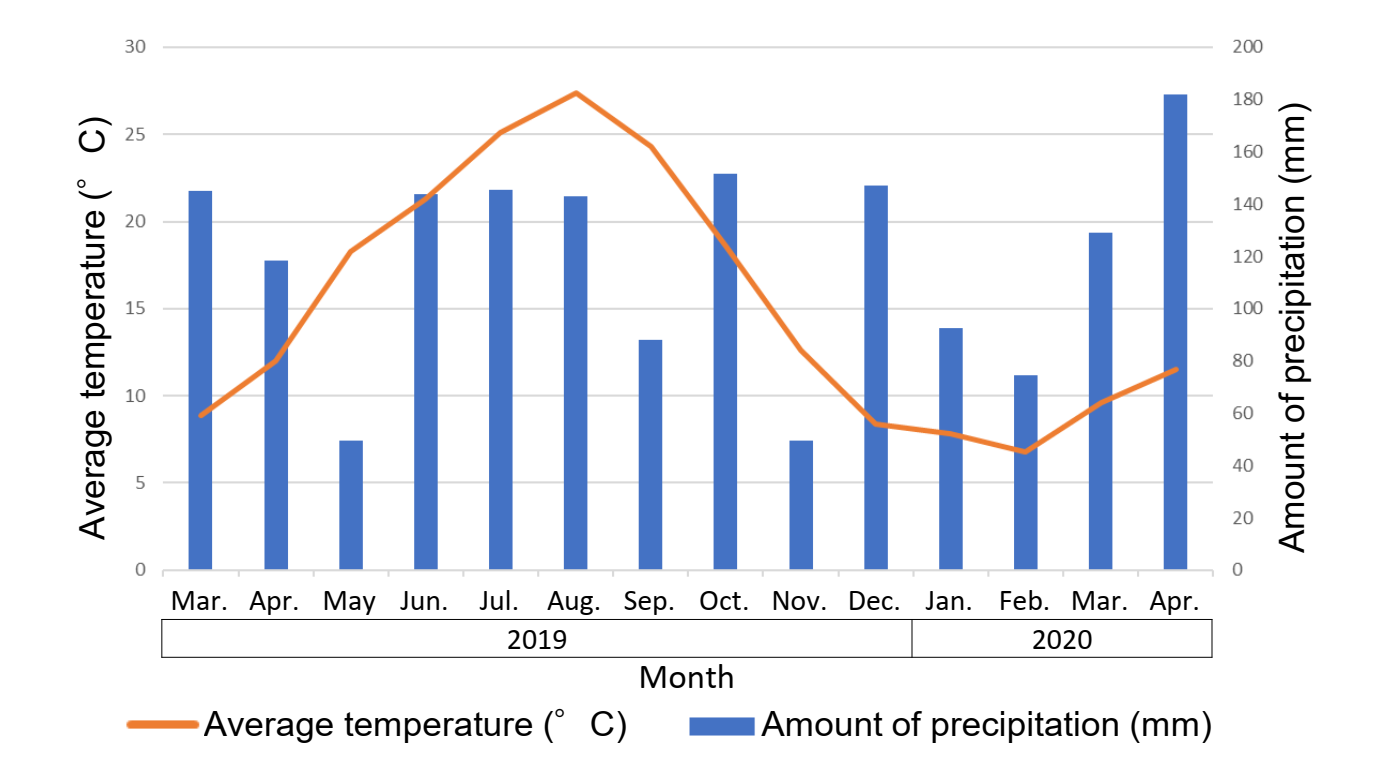


**Supplementary figure 1.**

**Weather information during the measurement period.**

The horizontal axis represents the month during the measurement period. The red line graph (left vertical axis) shows the daily average temperature, and the blue bar graph (right vertical axis) shows the total amount of precipitation.

**
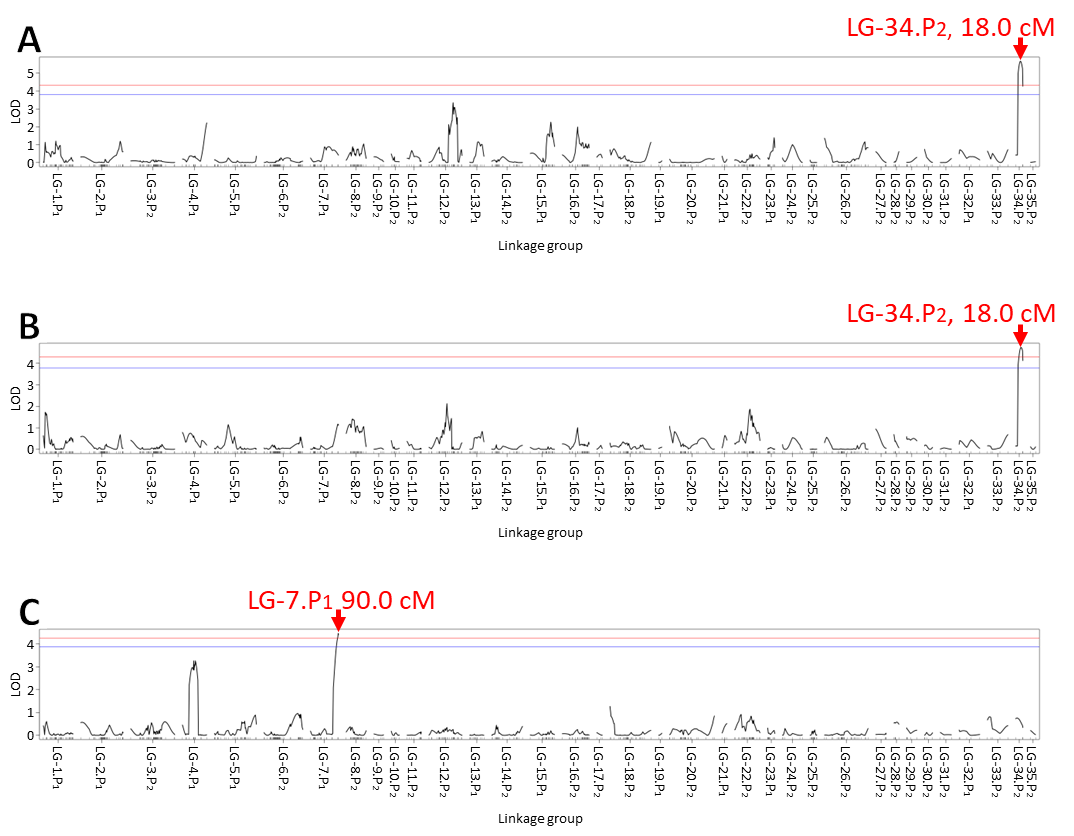
**

**Supplementary figure 2.**

**The LOD value of the trait with the highest LOD value on each measurement day and at each QTL.**

A is the LOD value of PC1 at the QTL identified in LG-34.P_2_ on April 16, 2019. B is the LOD values of VARI at the QTL identified in LG-34.P_2_ on April 9, 2020. C is the LOD values of SR at the QTL identified in LG-7.P_1_ on April 9, 2020. The vertical axis shows the LOD values, and the horizontal axis shows the chromosome position. Red arrows in the figure indicate the peak position.
